# Autoantibodies against eukaryotic translation elongation factor 1 delta in two patients with autoimmune cerebellar ataxia

**DOI:** 10.3389/fimmu.2023.1289175

**Published:** 2024-01-25

**Authors:** Liyuan Guo, Haitao Ren, Siyuan Fan, Xingchen Chao, Mange Liu, Hongzhi Guan, Jing Wang

**Affiliations:** ^1^ CAS Key Laboratory of Mental Health, Institute of Psychology, Chinese Academy of Sciences, Beijing, China; ^2^ Department of Psychology, University of Chinese Academy of Sciences, Beijing, China; ^3^ Department of Neurology, Peking Union Medical College Hospital, Chinese Academy of Medical Sciences and Peking Union Medical College, Beijing, China

**Keywords:** autoimmune, cerebellar ataxia, biomarker, novel autoantibody, anti-EEF1D antibody

## Abstract

**Background:**

Autoantibodies are useful biomarkers for the early detection and diagnosis of autoimmune cerebellar ataxia (ACA).

**Objective:**

To identify novel autoantibody candidates in ACA patients.

**Methods:**

Patients with cerebellar ataxia of unknown cause were recruited from July 2018 to February 2023. Anti-neural autoantibodies in patient samples were detected by tissue-based indirect immunofluorescence assay (TBA) on rat cerebellum sections. TBA-positive samples were further screened for well-established anti-neural autoantibodies using commercial kits. Tissue-immunoprecipitation (TIP) and subsequent mass spectrometric (MS) analysis were used to explore the target antigens of autoantibodies in samples that were TBA-positive but negative for known autoantibodies. The specific binding between autoantibodies and the identified target antigen was confirmed by neutralization experiments, recombinant cell-based indirect immunofluorescence assay (CBA), and western blotting experiments.

**Results:**

The eukaryotic translation elongation factor 1 delta (EEF1D) protein was identified as a target antigen of autoantibodies in samples from a 43-year-old female ACA patient, while the specific binding of autoantibodies and EEF1D was confirmed by subsequent experiments. A second anti-EEF1D autoantibody-positive ACA patient, a 59-year-old female, was detected in simultaneous screening. The main clinical manifestations in each of the two patients were cerebellar syndrome, such as unsteady walking and limb ataxia. Both patients received immunotherapy, including corticosteroids, intravenous immunoglobulin, and mycophenolate mofetil. Their outcomes provided evidence to support the effectiveness of immunotherapy, but the cerebellar atrophy that occurred before treatment may be irreversible.

**Conclusion:**

In the current study, we identified anti-EEF1D autoantibody as a novel autoantibody candidate in ACA. Its pathological roles and diagnostic value need to be further verified in larger-scale studies.

## 1 Introduction

Autoimmune cerebellar ataxia (ACA), which is an important cause of sporadic cerebellar syndrome and can lead to severe disability, is a potentially treatable disorder from which patients may recover with immunotherapy (1). Autoantibodies are useful biomarkers for the early detection and diagnosis of ACA.

Over the past decade, dozens of ACA-associated autoantibodies have been identified. Some well-characterized autoantibodies, such as autoantibodies against Yo, Hu, CV2, Ri, Ma2, and KLHL11, are considered etiological clues for paraneoplastic cerebellar degeneration (PCD) (2), while the pathological effects of many recently reported autoantibodies, such as autoantibodies against PED10A (3), AP3B2 (4), PRG5 (5), RGS8 (6), and Septin-5 (7), have not been fully studied. Although most ACA-related autoantibodies are rare and presumed to be non-pathogenic, a considerable proportion of autoantibody-positive patients are responsive to immunotherapy (8–11).

While a considerable number of ACA-related autoantigens have been identified, there are still many ACA patients whose serological profiles cannot be matched to any of the classified antigens. This limits the possibilities for diagnosis and treatment. In the current study, we detected novel autoantibody candidates in samples from two ACA patients and identified eukaryotic translation elongation factor 1 delta (EEF1D) as the target antigen.

## 2 Samples and methods

### 2.1 Samples

This study was approved by the Ethics Committee of Peking Union Medical College Hospital (PUMCH; JS-891 and JS-2184), and informed consent was obtained from each patient. From July 2018 to February 2023, patients who were diagnosed with cerebellar ataxia of unknown cause were enrolled in the study and registered with the program for encephalitis and paraneoplastic syndrome (PNS) at PUMCH. Serum samples from enrolled patients were tested using a tissue-based indirect immunofluorescence assay (TBA) on rat cerebellum sections (the method is described in section 2.2 below) and further screened for well-established anti-neural autoantibodies using commercial immunoblots or cell-based assay (CBA) test kits purchased from Euroimmun, Germany. Autoantibodies targeting MOG, AQP4, NMDA-R, CASPR2, AMPA-R, LGI1, GABAb-R, GAD-65, ITPR1, ZIC4, PKCγ, AP3B2, PCA-2, CARP VIII, Homer-3, NCDN, CV2/CRMP5, PNMA2, Ri, Yo, Hu, and amphiphysin were screened for. In total, 38 patients were TBA-positive and negative for the above autoantibodies. Additionally, 15 age- and sex-matched systematic autoimmune disease patients without neurological manifestations and 30 healthy donors were enrolled. Serum samples from 38 patients and three healthy controls were subjected to tissue immunoprecipitation (TIP) and combined mass spectrometry (MS). Serum samples from all subjects were tested for anti-EEF1D autoantibodies using a recombinant cell-based indirect immunofluorescence assay (CBA).

### 2.2 Tissue-based indirect immunofluorescence assay

TBA was performed as described by Guo et al. (12). Thirty microliters of PBS-diluted or undiluted samples (serum and cerebrospinal fluid (CSF)) from patients or controls was incubated with rat cerebellum section slides at 4°C for 3 h; fluorescein isothiocyanate (FITC)-labelled polyclonal goat anti-human IgG (Cat. ZF-0308, ZSBio, China) was then added and the sample was subsequently incubated at room temperature for 30 min. The sample was co-incubated with anti-EEF1D rabbit antibody (Cat. Ab153728, Abcam, USA) in the colocalization test, and Alexa Fluor 555-labelled goat anti-rabbit IgG antibody (Cat. Ab150078, Abcam, UK) was added together with FITC-labelled goat anti-human IgG. In the IgG subclass evaluation test, the binding of patient serum and rat cerebellum sections was performed as described above, followed by the secondary antibodies of unconjugated sheep anti-human IgG antibodies specific to IgG subclasses 1 to 4 (Nodics-Mubio, Netherlands, 1:100) and the tertiary antibodies of AF568-labelled donkey anti-sheep IgG (Invitrogen; absorbed against human IgG, 1:200). TBA results were examined using a DMi8 microscope (Leica, Germany) by two independent observers.

### 2.3 Tissue-immunoprecipitation and combined mass spectrometry

TIP was performed as described by Scharf et al. (13). The rat cerebellum was homogenized after shock-freezing in liquid nitrogen and then centrifuged at 13,000 × g at 4°C for 10 min. The supernatants were incubated with PBS-diluted serum at 4°C for 3 h. Protein G Dynabeads (Cat. 10003D, Thermo Fisher Scientific, USA) were then added, the mixture was rotated at 4°C overnight, and the immune complexes were eluted with 60 µl of Laemmli sample buffer (Cat. S3401, SIGMA, USA). The mixed eluate was directly analyzed using an Easy nLC 1000 (Thermo Fisher Scientific, USA) and Q Exactive Plus mass spectrometer (Thermo Fisher Scientific, USA) to obtain the amino acid sequence of the eluted proteins, as described by Guo et al. (12). The Proteome Discoverer 2.0 software package (Thermo Fisher Scientific, USA) was used to analyze the raw MS data, and corresponding proteins were identified according to *Rattus norvegicus* sequence data from the UniProt sequence database. Protein identification was supported by at least one unique peptide. The results were filtered based on a false discovery rate (FDR) of less than 1%. Quantitative comparisons of the proteome results for patients and controls were performed via label-free analysis with MaxQuant (14).

### 2.4 Recombinant expression of full-length EEF1D in HEK293 cells

HEK293 cells (CRL-1573, ATCC, VA, USA) were cultured in high-glucose DMEM (Cat. No. 11965092, Life Technologies, USA) supplemented with 10% FBS at 37°C and 5% CO_2_. A customized pcDNA3.1 plasmid containing full-length human EEF1D cDNA (NM_001130053) was purchased from GENEWIZ, China, and a 6x His-tag was added at the C-terminus. Transient transfection was performed using FuGene HD transfection reagent (Cat. E2311, Promega, USA). For CBA, cells were seeded on sterile cover glasses 24 hours before transfection; 48 hours after transfection, cells were fixed with 4% PFA. For western blot and neutralization experiments, transient transfection was performed in the same way, and total protein of transfected cells was obtained by lysis buffer (Cat. P0013D, Beyotime, China) 48 h later. The cell lysate was centrifuged at 13,000 × g at 4°C for 10 min, and the recombinant EEF1D proteins were roughly purified from the supernatant via Ni^2+^ affinity chromatography.

### 2.5 Western blot analysis

Roughly purified EEF1D proteins were electrophoresed on a 10% SDS-PAGE gel and then transferred to a PVDF membrane. The membrane was stained with Coomassie blue R-250 to represent total protein staining (15) and then blocked for 1 h in 5% non-fat milk and incubated with serum (1:20, PBS-diluted) from patients or healthy donors or anti-EEF1D rabbit antibody (1:2500, Cat. Ab153728, Abcam, USA). HRP-conjugated anti-human or anti-rabbit IgG (1:1000, Cat. A0201 and A0208, Beyotime, China) was used to detect the primary antibodies.

### 2.6 Neutralization experiments

Before TBA, the patient’s serum was diluted 1:1600 with PBS and preincubated with 1 µg of purified EEF1D protein at 4°C overnight.

### 2.7 Recombinant cell-based indirect immunofluorescence assay

Fixed His-tagged human full-length EEF1D-overexpressing HEK293 cells were incubated with serum or CSF and anti-His-tag mouse antibody (Cat. AH367, Beyotime, China) for 2 h at room temperature. FITC-labelled goat anti-human IgG antibodies (Cat. ZF-0308, ZSBio, China) were used to detect the binding of human IgG. Alexa Fluor 555-labelled goat anti-mouse IgG antibodies (Cat. Ab150114, Abcam, USA) and DAPI (Cat. C1002, Beyotime, China) were also added to visualize the EEF1D protein and cell nuclei. The slides were observed using a DMi8 microscope (Leica, Germany).

## 3 Results

### 3.1 Identification of the novel autoantigen EEF1D

**Figure 1:**
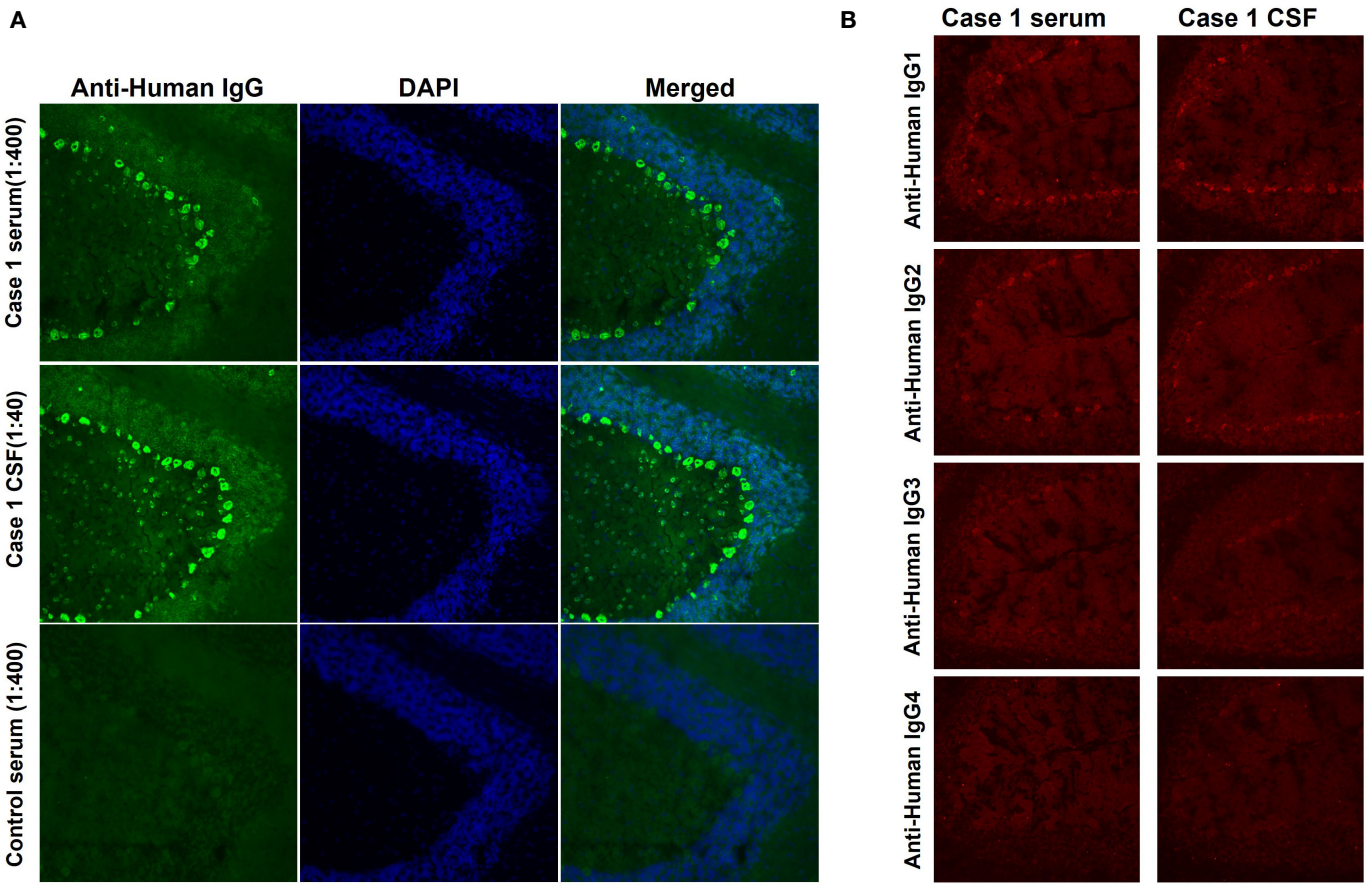
IgG immunofluorescence staining in rat cerebellum. **(A)** Serum (1:400 dilution) and CSF (1:40 dilution) from case 1 and serum from a control (1:40 dilution) were incubated with rat cerebellum sections. Fine IgG staining was obtained in both the Purkinje cell layer and the molecular cell layer (green). Nuclei were labelled by DAPI (blue). **(B)** The autoantibodies include subclasses of IgG1, IgG2, and IgG3, but not IgG4.

From July 2018 to November 2019, we recruited five ACA patients who were negative for established neural autoantibodies, one of whom was identified as positive for anti-Rab6a/b in our previous study (12). Serum and CSF samples from the other four patients, including case 1 in the current study, were used for further autoantigen identification. The serum and CSF of the patient in case 1 produced a specific IgG immunostaining pattern in neuronal soma staining, especially in the Purkinje cell layer and molecular layer of the rat cerebellum (**Figure 1A**). The IgG subclass of the autoantibodies includes IgG1, IgG2, and IgG3 (**Figure 1B**).

Immunocomplexes of the index patient serum and rat cerebellum were analyzed via MS. The protein EEF1D was identified as a candidate autoantigen since it did not appear in immunocomplexes precipitated in parallel by serum samples from three healthy donors. As shown in **Supplementary Figure S1**, the homology of rat Eef1d protein and human EEF1D is high (93%). The human EEF1D gene encodes different spliced protein isoforms; the long isoform (NP_115754) is only expressed in the brain and testis, and the shorter isoform (NP_001951), which is 100% similar to the C-terminus of the long isoform, is ubiquitously expressed. In rats, there are also isoforms of different lengths (UniProt ID: Q68FR9-1 and Q68FR9-2). The Proteome Discoverer 2.0 software package utilized Q68FR9-1 (the shorter one) to analyze peptides by default. As a result, 17 peptides were identified in EEF1D with more than 60% protein coverage (adjacent or overlapping peptides are merged in **Supplementary Figure S1**).

**Figure 2:**
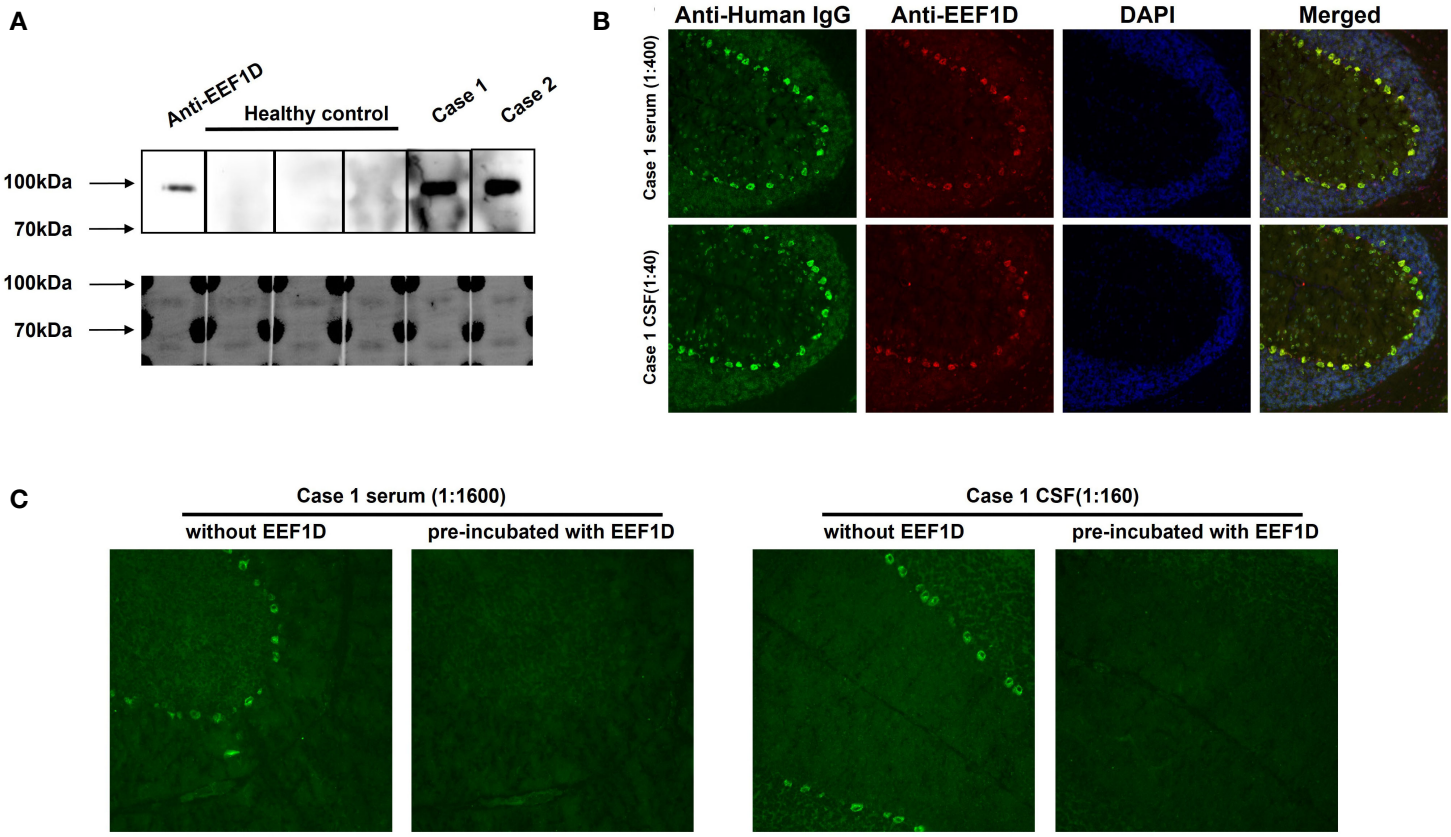
Specific binding between autoantibodies and human full-length EEF1D. **(A)** Reactivity of serum (1:20 dilution) with recombinant human EEF1D protein in case 1 and case 2 patients, but not in healthy controls. Upper: chemiluminescence; lower: Coomassie staining. **(B)** Specific binding between rat cerebellum tissue sections and case 1 serum (green) or anti-EEF1D antibody (red). The merged images show colocalization of the reactivity in the same region (yellow). Nuclei were labelled by DAPI (blue). **(C)** After preincubation with recombinant human EEF1D protein, the staining of case 1 samples was abolished.

Considering the brain-specific expression of the long isoform, we recombinantly expressed the full-length EEF1D cDNA for further detection. From August 2020 to February 2023, 33 other ACA patients who were TBA-positive but negative for known autoantibodies were enrolled. Among them, a second anti-EEF1D IgG-positive patient (case 2) was detected via CBA. As shown in **Figure 2A**, specific binding of EEF1D protein was detected in western blotting with serum or CSF from the patients in cases 1 and 2, but not with serum from healthy controls. In the rat cerebellum in TBA, the anti-EEF1D antibody performed neuronal soma staining, similar to the serum and CSF of case 1 (**Figure 2B**). After neutralization with human EEF1D protein, the staining of case 1 samples was abolished in the rat cerebellum in TBA (**Figure 2C**).

### 3.2 Specificity of anti-EEF1D autoantibodies in ACA patients

Fifteen systematic autoimmune disease patients without neurological manifestations and 30 healthy donors were enrolled for testing for anti-EEF1D autoantibodies, and none of them were positive for recombinant EEF1D CBA. As shown in **Supplementary Figure S2**, the serum and CSF of the patients in cases 1 and 2 showed positive staining, which was specifically distinguished them from serum samples from two healthy controls and one patient with systemic lupus erythematosus (control data other than these three are not shown).

### 3.3 Clinical data on anti-EEF1D-positive patients

#### 3.3.1 Case 1

**Figure 3:**
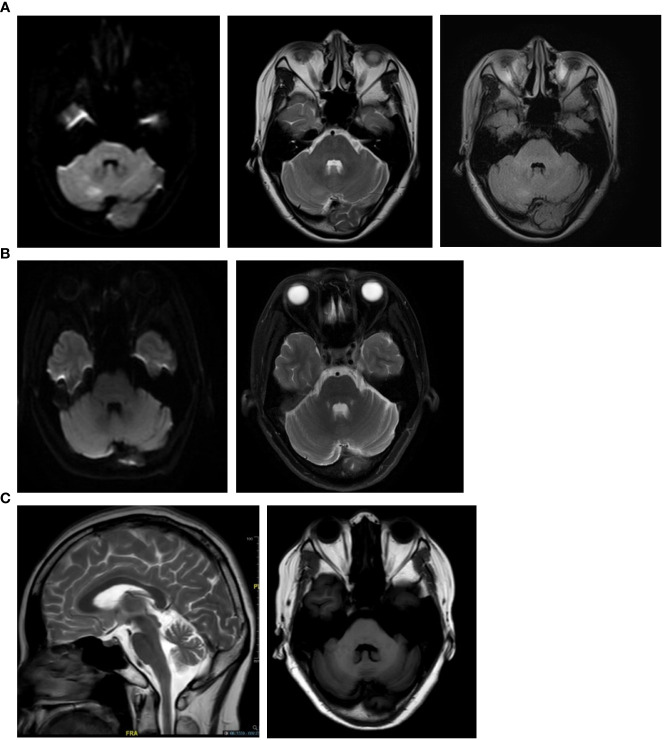
Brain magnetic resonance imaging of the anti-EEF1D-positive patient in case 1. **(A)** Brain MRI scans conducted two months after disease onset (May 2019) showed multiple hyperintense lesions in the cerebellum of the patient in case 1. **(B)** One month later (June 2019), follow-up brain MRI scans showed that the lesions in the cerebellum of the patient in case 1 had disappeared. **(C)** MRI conducted in October 2019 showed cerebellar atrophy.

A 43-year-old female presented in June 2019 with progressive dizziness, tinnitus, and unstable gait for 3 months. Her past medical history was noted for connective tissue disease, for which she was taking mycophenolate mofetil (MMF) and a low-dose corticosteroid. Physical examination showed a stumbling gait, unsteadiness in the heel-to-shin test, and decreased tendon reflexes in the left upper extremity. There was no dysarthria, nystagmus, or pathological reflex. The patient was positive for anti-SSA and anti-nuclear antibodies (ANA). Her CSF white blood cell count was 4/μL, while CSF-specific oligoclonal bands (SOBs) were present. Brain magnetic resonance imaging (MRI) performed in May 2019 showed multiple hyperintense lesions in DWI and FLAIR sequences, which disappeared 1 month later (**Figure 3**). A neuroconductive study revealed decreased sensory nerve action potentials in the bilateral median nerves and right ulnar nerve. Screening for infection, toxins, metabolic diseases, and malignancy was unremarkable. ACA with peripheral neuropathy was diagnosed, and the patient received pulse corticosteroid therapy. MMF treatment was maintained. Her symptoms improved transiently but then continued to deteriorate, and she complained of episodic clonic shaking of the left extremities. Brain MRI conducted in October 2019 revealed cerebellar atrophy. Upon video follow-up in January 2023, she was bedridden and obtained a Scale for Assessment and Rating of Ataxia (SARA) score of 23.

#### 3.3.2 Case 2

A 59-year-old female presented with unstable gait for 5 years and paresthesia in the bilateral calf for 2 weeks. Physical examination revealed gait and bilateral limb ataxia with a decreased tendon reflex in the lower extremities. The sensation was intact. The patient was also positive for anti-SSA and ANA. CSF examinations were unremarkable except for the presence of SOBs. Cerebellar atrophy was noted in brain MRI. Malignancy screening was negative. The patient was diagnosed with ACA and peripheral neuropathy. After pulse corticosteroid therapy, intravenous immunoglobulin, and MMF treatment, her symptoms were alleviated and remained stable for 3 years.

Clinical data on the two patients are summarized in **Table 1**.

**Table 1:** Clinical findings, therapies, and outcomes for two anti-EEF1D IgG-positive patients.

Patient, sex, age at onset	Clinical summary	Systemic autoantibodies/autoimmune diseases	Brain MRI	CSF findings	Immunotherapy	Follow-up time (months)	Outcome
Case 1 Female, 43	Subacute cerebellar syndrome with peripheral neuropathy	Anti-SSA, ANA, connective tissue disease	Multiple lesions in the cerebellum which disappeared 1 month later; cerebellar atrophy on follow-up	WBC 4/μL, SOB (+)	Corticosteroids, MMF	40	Worsened after transient improvement
Case 2 Female, 54	Chronic cerebellar syndrome with peripheral neuropathy	Anti-SSA, ANA	Cerebellar atrophy	WBC 2/μL, SOB (+)	IVIg, corticosteroids, MMF	33	Stabilized

ANA, anti-nuclear antibody; CSF, cerebrospinal fluid; IVIg, intravenous immunoglobulin; MMF, mycophenolate mofetil; OB, oligoclonal bands; WBC, white blood cells. (+), means positive.

## 4 Discussion

To date, approximately 30 types of ACA-related anti-cerebellar autoantibodies have been reported (16). Although most ACA-related autoantibodies are targeted at intracellular compositions and are regarded as non-pathogenetic (17), they serve as important clues for immune-mediated mechanisms, since they are specific to ACA and not detected in serum or CSF samples from patients with other systematic autoimmune diseases, those with ataxia of other etiology, or healthy subjects. Serological testing that targets specific autoantigens can aid in the diagnosis and treatment of patients with unexplained symptoms of cerebellar ataxia.

In the current study, we identified anti-EEF1D IgG antibodies in two ACA patients. The diagnosis was based on the inflammatory CSF profile, the presence of systematic autoantibodies, and the exclusion of other etiologies, according to the diagnostic criteria of the International Task Force (1). The subclasses of autoantibodies include IgG1, IgG2, and IgG3, similar to previously identified anti-neural antibodies (18).

EEF1D belongs to the eukaryotic translation elongation 1 complex, which is localized intracellularly and plays essential roles in aminoacyl-tRNA-related protein translation elongation (19, 20). The human EEF1D gene encodes at least four different splice isoforms: spatially, the longest isoform (647 aa) is specifically expressed in the brain and testis, and the other, shorter isoforms (252–281 aa) are ubiquitously expressed (21). Mutations in the EEF1D gene have been identified as causes of several neurodevelopmental disorders (21). Deletion of the long isoform in mice leads to seizures and abnormal behaviors (22). EEF1D has also been reported to be overexpressed in various tumors, such as glioblastoma, glioma, and lymphoma (23–26). Additionally, there are eight pseudogenes of EEF1D in the human genome, and EEF1D pseudogene-derived proteins or short peptides have been reported to be correlated with a range of tumors and neurological diseases, such as breast carcinoma, ankylosing spondylitis, non-small cell lung cancer, and lymphoma (27).

Since the EEF1D protein is an intracellular antigen, the pathogenic roles of autoantibodies against EEF1D require further study. The outcomes of the two patients reported on here provide evidence to support the effectiveness of immunotherapy. One patient experienced a brief remission of symptoms but worsened again soon after weaning from pulse corticosteroid therapy, suggesting the need for more intense and durable immunosuppressive treatment. The other patient stabilized after immunotherapy, which indicates that the aberrant autoimmune process might have been suppressed. However, the loss of cerebellar function, as implicated by cerebellar atrophy, might not be reversible. Therefore, it is important to start immunotherapy early, before non-restorable cerebellar damage has occurred, and to maintain adequate intensity and course (28). The similar clinical manifestations in both patients, with cerebellar ataxia as the main complaint, inflammation of CSF, and effectiveness of immunotherapy, suggest that anti-EEF1D autoantibodies can potentially be used as biomarkers in ACA. The discovery of anti-EEF1D antibodies will contribute to the timely diagnosis of ACA, which can hopefully improve patient outcomes.

In conclusion, we identified a new autoantibody candidate against the EEF1D protein in two ACA patients. Although its role in the mechanism of disease requires further study, our findings expand the spectrum of diagnostic anti-neuronal antibodies in ACA.

## Data availability statement

The original contributions presented in the study are included in the article/**Supplementary Material**. Further inquiries can be directed to the corresponding authors.

## Ethics statement

The studies involving humans were approved by Peking Union Medical College Hospital. The studies were conducted in accordance with the local legislation and institutional requirements. The participants provided their written informed consent to participate in this study. Written informed consent was obtained from the individual(s) for the publication of any potentially identifiable images or data included in this article.

## Author contributions

LG: Conceptualization, Methodology, Writing – original draft. HR: Methodology, Writing – original draft. SF: Methodology, Writing – review & editing. XC: Methodology, Writing – review & editing. ML: Methodology, Writing – review & editing. HG: Conceptualization, Supervision, Validation, Writing – review & editing. JW: Conceptualization, Supervision, Validation, Writing – review & editing.

## Supplementary material

Supplementary Figure 1 Sequence alignment between MS fragments and human/rat EEF1D protein. Peptide sequences were aligned with both long and short isoforms of the human/rat EEF1D protein.

Supplementary Figure 2 Distinguished CBA patterns of samples from anti-EEF1D-positive patients and healthy controls. In recombinant EEF1D CBA, samples from index patients were positive, and sera from two healthy controls (control 1 and control 2) and a systemic lupus erythematosus patient (control 3) were negative.
