# Supplementary figures and images for: Autoantibodies against eukaryotic translation elongation factor 1 delta in two patients with autoimmune cerebellar ataxia
Source: Front Immunol. 2024 Jan 25;14:1289175. doi: 10.3389/fimmu.2023.1289175 (PMC10850295; doi:10.3389/fimmu.2023.1289175)

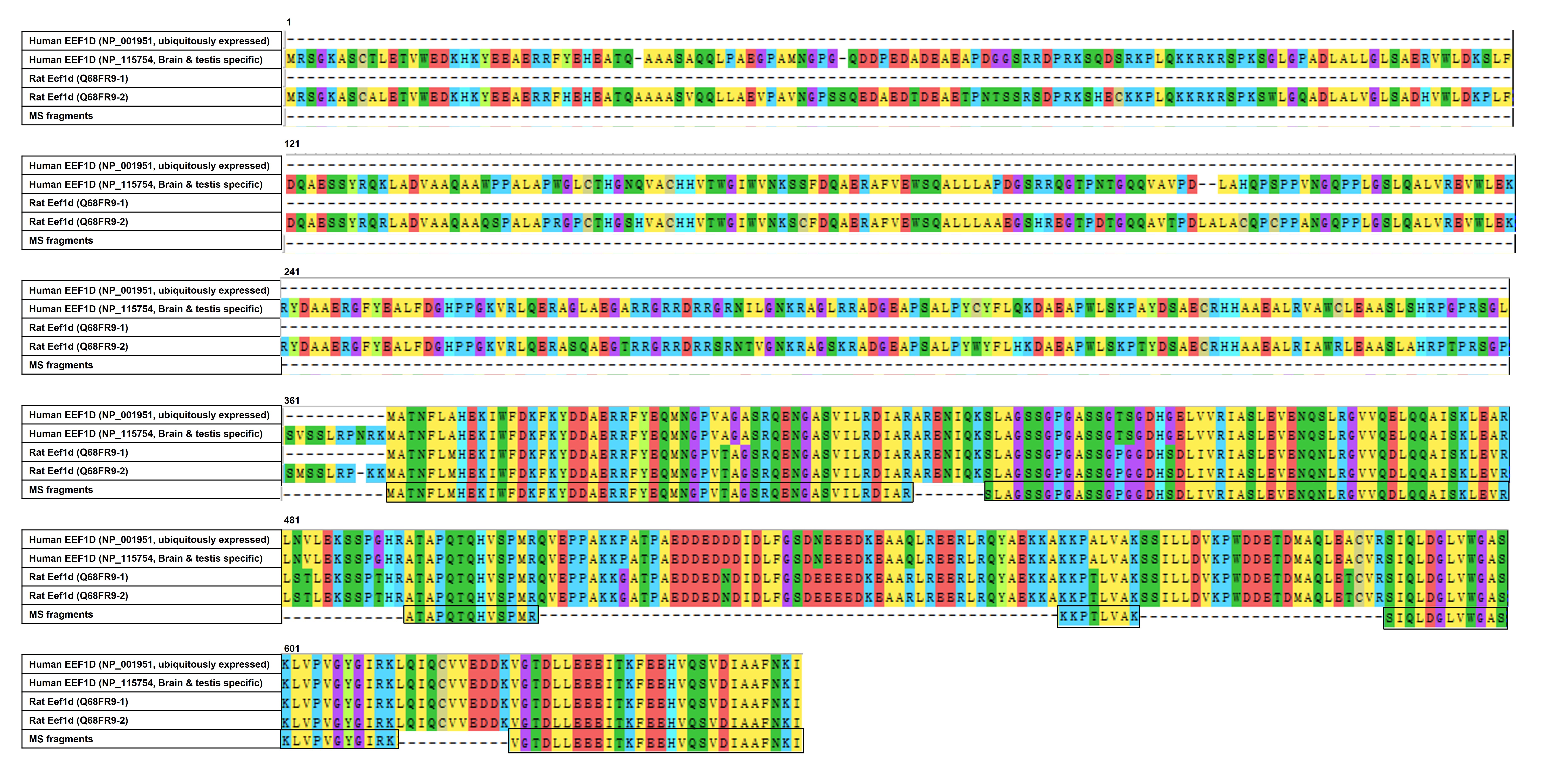

Supplement: Supplementary Figure 1 — Sequence alignment between MS fragments and human/rat EEF1D protein. Peptide sequences were aligned with both long and short isoforms of the human/rat EEF1D protein. [file Image_1.jpeg]

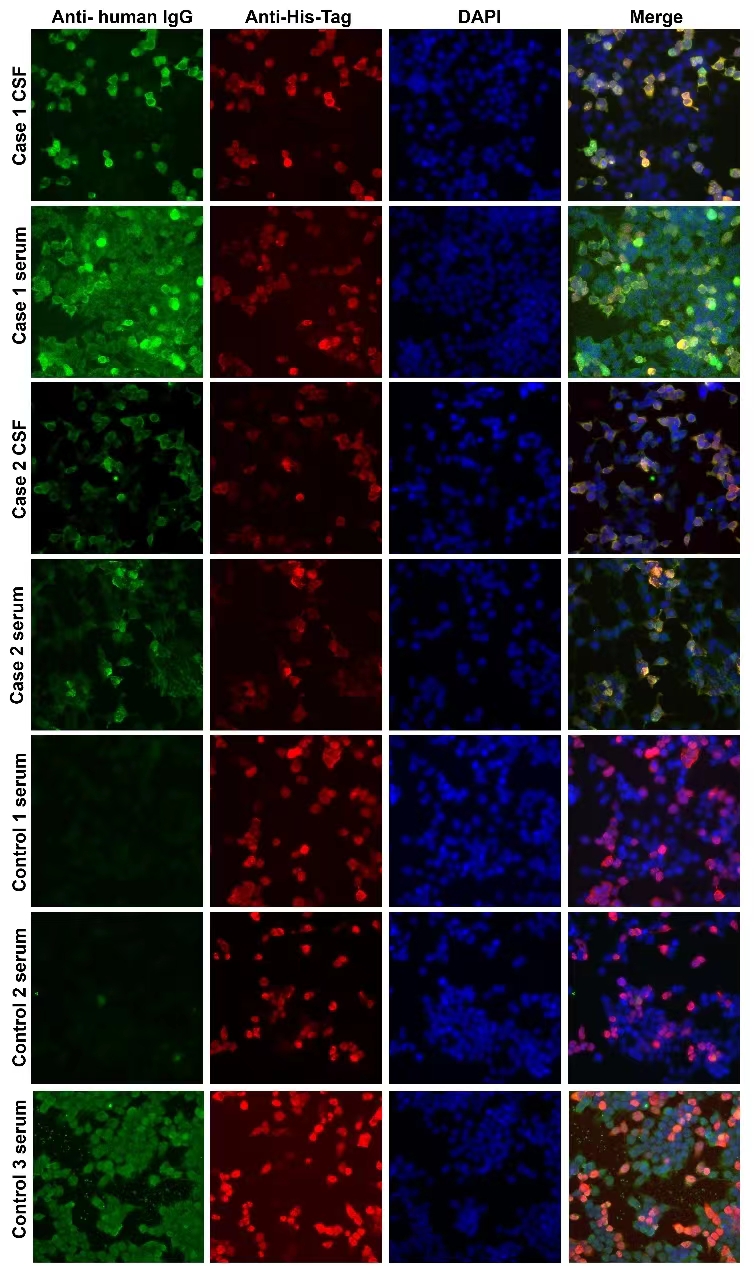

Supplement: Supplementary Figure 2 — Distinguished CBA patterns of samples from anti-EEF1D-positive patients and healthy controls. In recombinant EEF1D CBA, samples from index patients were positive, and sera from two healthy controls (control 1 and control 2) and a systemic lupus erythematosus patient (control 3) were negative. [file Image_2.jpeg]
